# Supplementary material for: Alu Deletions in LAMA2 and CDH4 Genes Are Key Components of Polygenic Predictors of Longevity
Source: Int J Mol Sci. 2022 Nov 4;23(21):13492. doi: 10.3390/ijms232113492 (PMC9657309; doi:10.3390/ijms232113492)
Supplement: Supplementary file 1 [file ijms-23-13492-s001.zip › ijms-1982379-supplementary.pdf]

**Supplementary Table S1.** Results of a pairwise comparative analysis of the genotype/alleles frequencies of the Alu-polymorphic locus Ya5ACE of *ACE* gene in the different age groups.

| Age group | Genotype/ allele | Young Adults | Middle-age Adults | Elderly | Old |
|---|---|---|---|---|---|
| Young | DD | – | – | – | – |
| | DI | – | – | – | – |
| | II | – | – | – | – |
| | D / I | – | – | – | – |
| Middle-aged | DD | 0.705 | – | – | – |
| | DI | 0.124 | – | – | – |
| | II | 0.145 | – | – | – |
| | D / I | 0.585 | – | – | – |
| Elderly | DD | 1.000 | 0.696 | – | – |
| | DI | 0.939 | 0.094 | – | – |
| | II | 0.928 | 0.132 | – | – |
| | D / I | 1.000 | 0.573 | – | – |
| Old seniors | DD | 0.887 | 0.605 | 0.940 | – |
| | DI | 0.604 | **0.028** | 0.732 | – |
| | II | 0.705 | 0.051 | 0.811 | – |
| | D / I | 0.891 | 0.471 | 0.961 | – |
| Long-livers | DD | 0.393 | 0.665 | 0.377 | 0.263 |
| | DI | 0.445 | 0.503 | 0.381 | 0.187 |
| | II | 1.000 | 0.195 | 1.000 | 0.787 |
| | D / I | 0.548 | 0.279 | 0.577 | 0.583 |

Here and in Tables 2-10: results in bold type correspond to P<0.05

**Supplementary Table S2.** Results of a pairwise comparative analysis of the genotype/alleles frequencies of the Alu-polymorphic locus Ya5NBC182 of *HECW1* gene in the different age groups.

| Age group | Genotype/ allele | Young Adults | Middle-age Adults | Elderly | Old |
|---|---|---|---|---|---|
| Young | DD | – | – | – | – |
| | DI | – | – | – | – |
| | II | – | – | – | – |
| | D / I | – | – | – | – |
| Middle-aged | DD | **0.016** | – | – | – |
| | DI | 0.226 | – | – | – |
| | II | 0.826 | – | – | – |
| | D / I | 0.190 | – | – | – |
| Elderly | DD | **0.003** | 1.000 | – | – |
| | DI | 0.368 | 0.591 | – | – |
| | II | 0.319 | 0.589 | – | – |
| | D / I | **0.029** | 0.698 | – | – |
| Old seniors | DD | 0.126 | 0.192 | 0.081 | – |
| | DI | 0.228 | 0.691 | 0.819 | – |
| | II | 0.873 | 0.624 | 0.148 | – |
| | D / I | 0.589 | 0.305 | **0.044** | – |
| Long-livers | DD | 0.853 | 0.073 | **0.029** | 0.315 |
| | DI | 1.000 | 0.281 | 0.476 | 0.350 |
| | II | 0.917 | 0.905 | 0.473 | 0.781 |
| | D / I | 0.874 | 0.289 | 0.087 | 0.835 |

**Supplementary Table S3.** Results of a pairwise comparative analysis of the genotype/alleles frequencies of the Alu-polymorphic locus Yb8NBC597 of *SEMA6A* gene in the different age groups.

| Age group | Genotype/ allele | Young Adults | Middle-age Adults | Elderly | Old |
|---|---|---|---|---|---|
| Young | DD | – | – | – | – |
| | DI | – | – | – | – |
| | II | – | – | – | – |
| | D / I | – | – | – | – |
| Middle-aged | DD | 0.275 | – | – | – |
| | DI | 0.145 | – | – | – |
| | II | 0.412 | – | – | – |
| | D / I | 0.518 | – | – | – |
| Elderly | DD | 0.204 | 1.000 | – | – |
| | DI | 0.092 | 0.908 | – | – |
| | II | 0.377 | 1.000 | – | – |
| | D / I | 0.487 | 1.000 | – | – |
| Old seniors | DD | 0.752 | 0.373 | 0.275 | – |
| | DI | 0.292 | 0.410 | 0.371 | – |
| | II | 0.154 | 1.000 | 0.740 | – |
| | D / I | 0.843 | 0.408 | 0.294 | – |
| Long-livers | DD | 0.368 | 0.063 | **0.035** | 0.178 |
| | DI | 0.757 | 0.273 | 0.292 | 0.641 |
| | II | **0.006** | 0.138 | 0.053 | 0.077 |
| | D / I | 0.059 | **0.029** | **0.010** | 0.055 |

**Supplementary Table S4.** Results of a pairwise comparative analysis of the genotype/alleles frequencies of the Alu-polymorphic locus Yb8NBC516 of *CDH4* gene in the different age groups.

| Age group | Genotype/ allele | Young Adults | Middle-age Adults | Elderly | Old |
|---|---|---|---|---|---|
| Young | DD | – | – | – | – |
| | DI | – | – | – | – |
| | II | – | – | – | – |
| | D / I | – | – | – | – |
| Middle-aged | DD | 0.166 | – | – | – |
| | DI | 1.000 | – | – | – |
| | II | 0.204 | – | – | – |
| | D / I | 0.075 | – | – | – |
| Elderly | DD | 0.799 | 0.233 | – | – |
| | DI | 1.000 | 1.000 | – | – |
| | II | 0.762 | 0.317 | – | – |
| | D / I | 0.719 | 0.139 | – | – |
| Old seniors | DD | 0.286 | **0.011** | 0.143 | – |
| | DI | 0.387 | 0.579 | 0.408 | – |
| | II | 0.931 | 0.175 | 0.802 | – |
| | D / I | 0.615 | **0.019** | 0.336 | – |
| Long-livers | DD | 0.070 | 0.886 | 0.105 | **0.001** |
| | DI | 0.591 | 0.702 | 0.602 | 0.927 |
| | II | **0.027** | 0.584 | 0.052 | **0.015** |
| | D / I | **0.008** | 0.653 | **0.018** | **$3\times10^{-4}$*** |

Here and in Tables 10: * indicates P-value that retain statistical significance after adjusting for multiple comparisons ($P_{Bonf}<0.05$).

**Supplementary Table S5.** Results of a pairwise comparative analysis of the genotype/alleles frequencies of the Alu-polymorphic locus Ya5ac2145 of *STK38L* gene in the different age groups.

| Age group | Genotype/ allele | Young Adults | Middle-age Adults | Elderly | Old |
|---|---|---|---|---|---|
| Young | DD | – | – | – | – |
| | DI | – | – | – | – |
| | II | – | – | – | – |
| | D / I | – | – | – | – |
| Middle-aged | DD | 0.691 | – | – | – |
| | DI | 0.678 | – | – | – |
| | II | 1.000 | – | – | – |
| | D / I | 0.719 | – | – | – |
| Elderly | DD | 0.822 | 0.894 | – | – |
| | DI | 0.722 | 1.000 | – | – |
| | II | 0.773 | 1.000 | – | – |
| | D / I | 1.000 | 0.812 | – | – |
| Old seniors | DD | 0.847 | 0.558 | 0.636 | – |
| | DI | 0.765 | 0.467 | 0.375 | – |
| | II | 0.776 | 0.732 | 0.426 | – |
| | D / I | 0.930 | 0.670 | 0.864 | – |
| Long-livers | DD | 0.398 | 0.220 | 0.231 | 0.395 |
| | DI | 0.262 | 0.157 | 0.104 | 0.323 |
| | II | 0.704 | 0.663 | 0.326 | 0.739 |
| | D / I | 0.512 | 0.381 | 0.514 | 0.560 |

**Supplementary Table S6.** Results of a pairwise comparative analysis of the genotype/alleles frequencies of the Alu-polymorphic locus Yb8AC702 of *PKHD1L1* gene in the different age groups.

| Age group | Genotype/ allele | Young Adults | Middle-age Adults | Elderly | Old |
|---|---|---|---|---|---|
| Young | DD | – | – | – | – |
| | DI | – | – | – | – |
| | II | – | – | – | – |
| | D / I | – | – | – | – |
| Middle-aged | DD | 0.624 | – | – | – |
| | DI | 0.225 | – | – | – |
| | II | 0.411 | – | – | – |
| | D / I | 0.831 | – | – | – |
| Elderly | DD | 0.401 | 0.902 | – | – |
| | DI | **0.032** | 0.618 | – | – |
| | II | 0.104 | 0.630 | – | – |
| | D / I | 0.674 | 0.890 | – | – |
| Old seniors | DD | 0.125 | 0.055 | **0.007** | – |
| | DI | 0.940 | 0.182 | **0.013** | – |
| | II | 0.086 | 0.661 | 0.930 | – |
| | D / I | 0.045 | 0.166 | 0.122 | – |
| Long-livers | DD | 0.067 | **0.030** | **0.005** | 0.470 |
| | DI | 0.639 | 0.112 | **0.013** | 0.516 |
| | II | 0.182 | 0.795 | 0.910 | 1.000 |
| | D / I | 0.046 | 0.117 | 0.108 | 0.645 |

**Supplementary Table S7.** Results of a pairwise comparative analysis of the genotype/alleles frequencies of the Alu-polymorphic locus Ya5ac2013 of *TEAD1* gene in the different age groups.

| Age group | Genotype/ allele | Young Adults | Middle-age Adults | Elderly | Old |
|---|---|---|---|---|---|
| Young | DD | – | – | – | – |
| | DI | – | – | – | – |
| | II | – | – | – | – |
| | D / I | – | – | – | – |
| Middle-aged | DD | 0.558 | – | – | – |
| | DI | 0.467 | – | – | – |
| | II | 0.819 | – | – | – |
| | D / I | 0.826 | – | – | – |
| Elderly | DD | 0.922 | 0.650 | – | – |
| | DI | 0.797 | 0.314 | – | – |
| | II | 0.700 | 0.500 | – | – |
| | D / I | 0.717 | 0.943 | – | – |
| Old seniors | DD | 0.603 | 0.835 | 0.684 | – |
| | DI | 0.644 | 0.708 | 0.423 | – |
| | II | 1.000 | 0.757 | 0.625 | – |
| | D / I | 0.744 | 1.000 | 0.959 | – |
| Long-livers | DD | 0.735 | 0.376 | 0.589 | 0.331 |
| | DI | 0.846 | 0.656 | 0.639 | 0.863 |
| | II | 0.522 | 0.809 | 0.295 | 0.448 |
| | D / I | 0.492 | 0.434 | 0.288 | 0.274 |

**Supplementary Table S8.** Results of a pairwise comparative analysis of the genotype/alleles frequencies of the Alu-polymorphic locus TPA25 of *PLAT* gene in the different age groups.

| Age group | Genotype/ allele | Young Adults | Middle-age Adults | Elderly | Old |
|---|---|---|---|---|---|
| Young | DD | – | – | – | – |
| | DI | – | – | – | – |
| | II | – | – | – | – |
| | D / I | – | – | – | – |
| Middle-aged | DD | 0.921 | – | – | – |
| | DI | 0.195 | – | – | – |
| | II | 0.092 | – | – | – |
| | D / I | 0.264 | – | – | – |
| Elderly | DD | 0.535 | 0.479 | – | – |
| | DI | 0.870 | 0.136 | – | – |
| | II | 0.353 | **0.012** | – | – |
| | D / I | 0.299 | **0.041** | – | – |
| Old seniors | DD | 0.820 | 0.719 | 0.643 | – |
| | DI | 0.482 | 0.405 | 0.355 | – |
| | II | 0.618 | 0.155 | 0.098 | – |
| | D / I | 0.921 | 0.287 | 0.175 | – |
| Long-livers | DD | 0.704 | 0.832 | 0.331 | 0.494 |
| | DI | 0.417 | 0.056 | 0.526 | 0.128 |
| | II | 0.607 | **0.041** | 0.762 | 0.262 |
| | D / I | 0.950 | 0.287 | 0.374 | 0.865 |

**Supplementary Table S9.** Results of a pairwise comparative analysis of the genotype/alleles frequencies of the Alu-polymorphic locus Ya5ac1986 of *COL13A1* gene in the different age groups.

| Age group | Genotype/ allele | Young Adults | Middle-age Adults | Elderly | Old |
|---|---|---|---|---|---|
| Young | DD | – | – | – | – |
| | DI | – | – | – | – |
| | II | – | – | – | – |
| | D / I | – | – | – | – |
| Middle-aged | DD | 0.093 | – | – | – |
| | DI | 0.685 | – | – | – |
| | II | 0.695 | – | – | – |
| | D / I | 0.303 | – | – | – |
| Elderly | DD | 0.329 | 0.397 | – | – |
| | DI | 0.180 | 0.117 | – | – |
| | II | 0.070 | 0.292 | – | – |
| | D / I | 0.065 | 0.652 | – | – |
| Old seniors | DD | 0.477 | 0.196 | 0.692 | – |
| | DI | 0.883 | 0.531 | 0.157 | – |
| | II | 0.567 | 1.000 | 0.112 | – |
| | D / I | 0.452 | 0.578 | 0.152 | – |
| Long-livers | DD | 0.122 | 0.864 | 0.530 | 0.247 |
| | DI | 0.399 | 0.747 | **0.030** | 0.258 |
| | II | 1.000 | 0.681 | 0.094 | 0.585 |
| | D / I | 0.510 | 0.683 | 0.297 | 1.000 |

**Supplementary Table S10.** Results of a pairwise comparative analysis of the genotype/alleles frequencies of the Alu-polymorphic locus Ya5-MLS19 of *LAMA2* gene in the different age groups.

| Age group | Genotype/ allele | Young Adults | Middle-age Adults | Elderly | Old |
|---|---|---|---|---|---|
| Young | DD | – | – | – | – |
| | DI | – | – | – | – |
| | II | – | – | – | – |
| | D / I | – | – | – | – |
| Middle-aged | DD | 0.135 | | | |
| | DI | 0.161 | | | |
| | II | 1.000 | | | |
| | D / I | 0.290 | | | |
| Elderly | DD | 0.338 | 0.498 | | |
| | DI | 0.746 | 0.267 | | |
| | II | 0.547 | 0.568 | | |
| | D / I | 0.326 | 0.895 | | |
| Old seniors | DD | **0.027** | 0.932 | 0.252 | |
| | DI | 0.291 | 0.506 | 0.491 | |
| | II | 0.180 | 0.246 | 0.595 | |
| | D / I | **0.021** | 0.440 | 0.256 | |
| Long-livers | DD | 0.200 | **0.010** | **0.029** | **$5\times10^{-4}$** |
| | DI | **0.010** | **$2\times10^{-4}$*** | **0.003** | **$6\times10^{-5}$*** |
| | II | 0.072 | 0.099 | 0.213 | 0.416 |
| | D / I | 0.851 | 0.442 | 0.458 | 0.061 |

**Supplementary Table S11.** Estimation of the individual ORs for elements of the identified polygenic predictors of longevity.

| Genotype/ allele | P | OR | $CI_{OR}$ |
|---|---|---|---|
| Comparable age periods – 18-74 | | | |
| *CDH4* Yb8NBC516*D | 0.033 | 1.509 | 1.033-2.204 |
| *LAMA2* Ya5-MLS19*ID | $2.081\times10^{-4}$ | 1.781 | 1.313-2.415 |
| *SEMA6A* Yb8NBC597*I | 0.049 | 1.406 | 1.001-1.974 |
| Comparable age periods – 18-89 | | | |
| *CDH4* Yb8NBC516*D | 0.014 | 1.565 | 1.094-2.239 |
| *CDH4* Yb8NBC516*DD | 0.006 | 1.709 | 1.162-2.513 |
| *LAMA2* Ya5-MLS19*ID | $4.246\times10^{-5}$ | 1.833 | 1.372-2.451 |
| *HECW1* Ya5NBC182*D | 0.945 | 0.988 | 0.703-1.388 |
| Comparable age periods – 60-89 | | | |
| *LAMA2* Ya5-MLS19*ID | $6.417\times10^{-5}$ | 1.841 | 1.365-2.483 |
| *HECW1* Ya5NBC182*I | 0.110 | 1.612 | 0.897-2.897 |
| Comparable age periods – 75-89 | | | |
| *CDH4* Yb8NBC516*DD | 0.001 | 2.106 | 1.365-3.249 |
| *LAMA2* Ya5-MLS19*D | 0.364 | 1.210 | 0.802-1.826 |
| *HECW1* Ya5NBC182*I | 0.741 | 1.063 | 0.739-1.531 |
| *ACE* Ya5ACE*I | 0.234 | 1.234 | 0.873-1.744 |

Note. P – P-value; OR – odds ratio; $CI_{OR}$ – 95% confidence interval for OR.
